# A modular design approach to polymer-coated ZnO nanocrystals

**DOI:** 10.1016/j.isci.2022.105759

**Published:** 2022-12-14

**Authors:** Elżbieta Chwojnowska, Justyna Grzonka, Iwona Justyniak, Tomasz Ratajczyk, Janusz Lewiński

**Affiliations:** 1 Institute of Physical Chemistry, Polish Academy of Sciences, Kasprzaka 44/52, 01-224 Warsaw, Poland; 2 Faculty of Materials Science and Engineering, Warsaw University of Technology, Wołoska 141, 02-507 Warsaw, Poland; 3 Faculty of Chemistry, Warsaw University of Technology, Noakowskiego 3, 00-664 Warsaw, Poland

**Keywords:** Nanocomplex, Nanotechnology, Nanostructure

## Abstract

Hybrid materials based on inorganic nanocrystals with organic polymers feature peculiar and fascinating properties and various applications. However, there is still a need for simple synthesis procedures that provide precise control over the polymer/nanocrystal microstructure of these materials. Herein, a novel organometallic approach to polymer-coated ZnO nanocrystals was developed. The presented method merges the initial ring-opening polymerization of ϵ-caprolactone mediated by an organozinc alkoxide initiator and an air-promoted transformation of the resulting macromolecular organozinc species. This one-pot procedure results in quantum-sized ZnO crystals with a core diameter of ca 3 nm coated by poly(ϵ-caprolactone) covalently bonded to the surface. Overall, the ability to create well-defined hybrid composites should provide a unique ability to access various nanosystems.

## Highlights

• One-pot organometallic procedure for preparing polymer-coated zinc oxide nanocrystals
• Alkylzinc aminoalcoholate as an initiator for ring-opening polymerization of caprolactone
• Macromolecular organozinc species as precursors for quantum-sized ZnO nanocrystals

## Graphical abstract

**Figure:**
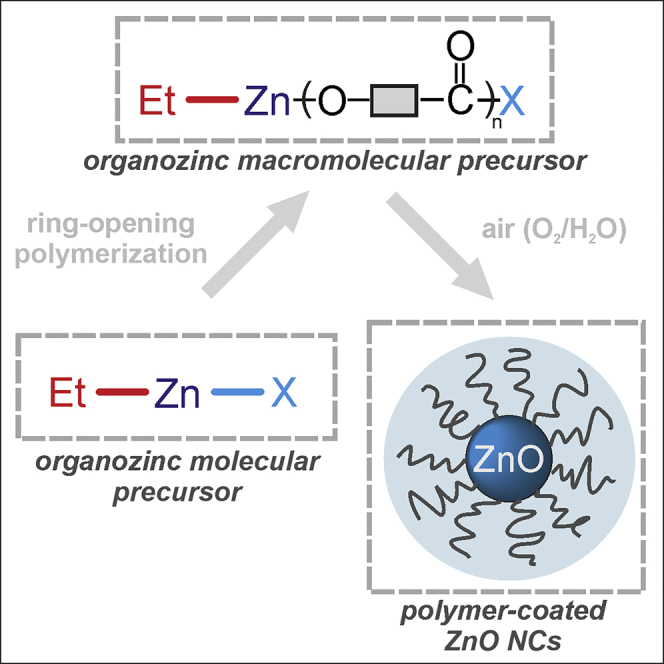

## Introduction

Synergic combination of inorganic nanocrystals (NCs) with organic polymers has received growing interest because the resulting hybrid materials feature peculiar and fascinating properties attractive for a wide variety of applications.^1^^,^^2^^,^^3^ Various strategies for preparing polymer-based nanocomposites have been reported over the last two decades.^3^^,^^4^^,^^5^^,^^6^^,^^7^ One of the simplest and widely used methods involves the direct mixing of NCs with a polymeric material.^4^^,^^5^^,^^6^^,^^7^ In this approach, the NCs surface is often modified prior to mixing to improve mixing ability and control the dispersion homogeneity of inorganic particles over the entire polymer matrix. To overcome the phase separation problem in nanocomposites, other methodologies, such as *in situ* growth of inorganic NCs in the polymer matrix, polymerization of monomers in the presence of separately obtained NCs, or modification of NCs surface involving covalent bonding of polymer chains to the surface are used.^4^^,^^5^^,^^6^^,^^7^^,^^8^^,^^9^^,^^10^^,^^11^ The latter methodology usually involves coupling the pre-synthesized polymer to surface groups previously introduced onto the NCs surface or radical polymerization directly from the NCs surface (via low-molecular-weight initiating groups on the surface or polymerizable surface ligands). All these approaches have been widely used for the preparation of a wide variety of zinc oxide (ZnO) NCs/polymer composites.^8^^,^^9^^,^^10^^,^^11^ For example, polymer coating allows obtaining water-soluble polymer-coated ZnO NCs essential for bioimaging.^12^^,^^13^^,^^14^ Moreover, ZnO NCs based polymeric nanocomposites have many applications in optoelectronics,^15^^,^^16^ water purification,^17^ antimicrobial packaging,^15^^,^^18^^,^^19^^,^^20^^,^^21^ and wound dressing.^22^^,^^23^ Additionally, it has been demonstrated that ZnO NCs effectively enhance the mechanical performance of polymers and their UV-shielding properties.^20^^,^^24^^,^^25^^,^^26^ Remarkably, in most published works on ZnO NCs/polymer composites, the common inorganic sol-gel procedure was used to prepare the parent NCs. This relatively cheap and fast manufacturing method has some significant limitations associated with, for example, low reproducibility and the lack of compositional consistency and stability of the inorganic core-organic shell interface of ZnO NCs in the quantum confinement regime.^27^^,^^28^^,^^29^ Recently, wet-organometallic approaches to prepare high-quality ZnO NCs^29^^,^^30^^,^^31^^,^^32^^,^^33^^,^^34^ have emerged as a promising alternative to sol-gel procedures. While organometallic methods are more time-consuming and require a more specialized set of tools, their superiority over the classical inorganic wet-chemical process for the preparation of stable and well-passivated quantum-sized ZnO crystals has already been well-documented, e.g. organometallic-derived ZnO NCs have appeared relatively bio-safe,^35^ prone to the ligand shell functionalization using the classic copper(I)-catalyzed click chemistry with the preservation of their photoluminescence properties,^36^ and highly prospective for applications in photocatalysis^37^ and photovoltaics as very effective electron transport material for perovskite solar cells.^38^ In turn that examples of nanocomposites involving ZnO NCs derived from an organometallic method are still scant.^11^^,^^32^^,^^33^

In the course of our systematic studies on the development of synthetic procedures for ZnO-based nanostructures, we have elaborated a general and convenient one-pot self-supporting organometallic method (OSSOM) based on the controlled exposure of RZnX type (R = alkyl; X = monoanionic organic ligand) precursors to air at ambient temperature.^29^^,^^39^^,^^40^^,^^41^ The OSSOM process is relatively slow in comparison to the classical inorganic sol-gel approach, however it allows access to unprecedented high-quality X-ligand-coated ZnO NCs and the unique option to adjust the physical properties of ZnO NCs such as stability, solubility, size control, aggregation degree, and long-lived luminescence. It provides the NCs with a uniquely passivated surface,^29^ allowing post-synthetic modification^36^^,^^37^ and the application of organozinc complexes incorporating chiral aminoalkoxide ligands paved the way for chiroptically active ZnO quantum dots.^40^ Based on our experiences in both the OSSOM procedure and the rational design of initiators for heterocyclic esters polymerization,^42^^,^^43^^,^^44^ herein we describe a conceptually different strategy for the preparation of polymer-coated ZnO NCs based on a one-pot two-step organometallic procedure combining the ring-opening polymerization and the OSSOM method.

## Results and discussion

**Figure 1:**
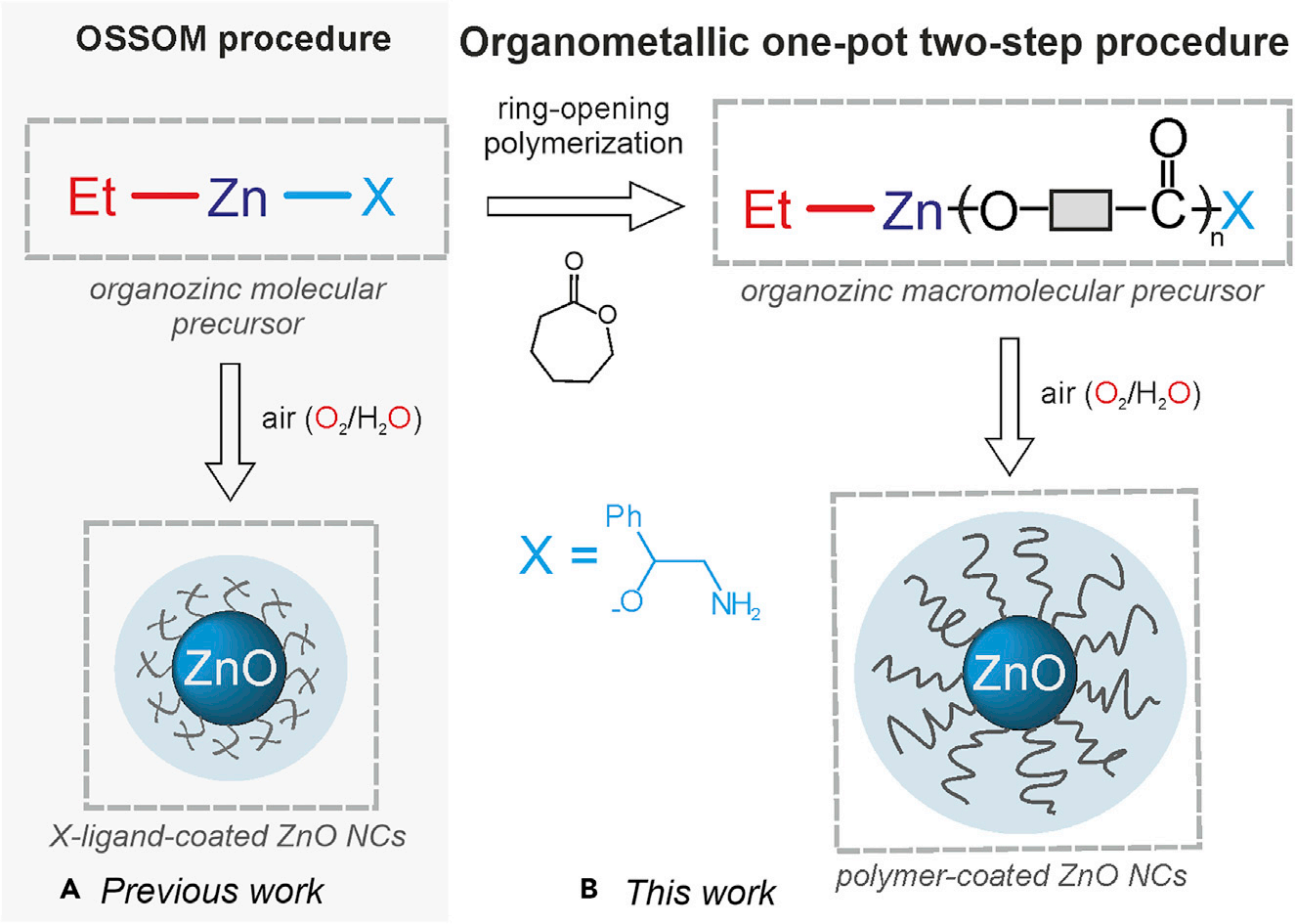
Representation of the OSSOM procedure and the organometallic one-pot two-step procedure leading to polymer-coated ZnO NCs (A) The general one-pot self-supporting organometallic procedure (OSSOM) based on the controlled exposure of an organometallic (EtZn-X) precursor to air at ambient temperature resulting in monoanionic-organic-ligand-coated ZnO NCs. (B) The organometallic one-pot two-step procedure: In the first step, polymers incorporating ethylzinc moieties are obtained through *in situ* ring-opening polymerization with ethylzinc 2-amino-1-phenylethanolate as an EtZn-X initiator. In the second step, so generated organometallic macromolecular EtZn(PCL-X) precursor is exposed to air resulting in polymer-coated ZnO NCs.

As mentioned above, we successfully employed organozinc complexes supported by chiral aminoalcoholate ligands, as the RZn-X type precursors of a series of chirotopically active ZnO quantum dots.^40^ (Figure 1) Keeping in mind that the application of zinc alkoxides supported by a wide variety of monoanionic organic ligands in the ring-opening polymerization (ROP) of cyclic esters is conceptually straightforward,^45^^,^^46^^,^^47^^,^^48^^,^^49^ we wondered whether it might be possible to use alkylzinc aminoalcoholates as suitable initiators for the preparation of polyesters anchored to an organometallic center, which in turn can be used as a macromolecular RZn(polyester-X) type precursor of ZnO NCs according to the OSSOM procedure. Results from the first proof of concept studies are reported later in discussion.

**Figure 2:**
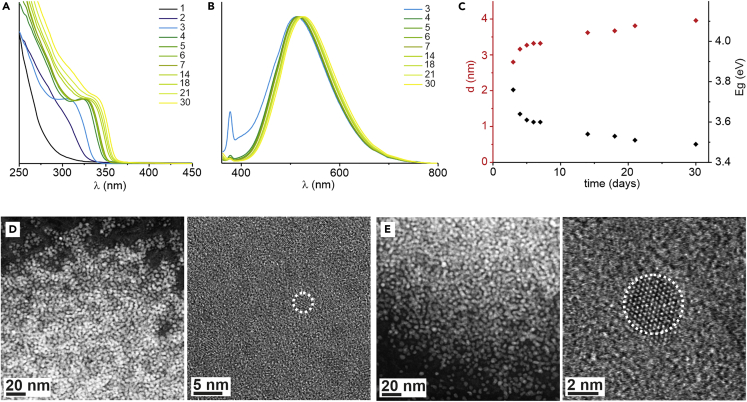
The spectroscopic and HRTEM characterization of ZnO-PCL (A and B) Normalized (A) UV–Vis absorption and (B) photoluminescence spectra of ZnO-PCL. Different colors and numbers indicate the number of days of exposure to air. (C) Band gaps from absorption measurements (black) and particle size calculated from Brus formula (blue). (D and E) HRTEM images of ZnO-PCL after (D) 5 and (E) 30 days of exposure to air.

Initially, we synthesized ethylzinc 2-amino-1-phenylethanolate (EtZn-X) as a model compound and, in a control experiment, tested its catalytic activity as an initiator in the ring-opening polymerization of ϵ-caprolactone (CL). Promisingly, the alkoxide EtZn-X was found to efficiently initiate the polymerization of CL in tetrahydrofuran (THF) at room temperature to give poly(ϵ-caprolactone) (PCL) bearing the anticipated aminoalcoholate and ethylzinc end-groups, [EtZn(PCL-X)]. Moreover, after the standard workup (removing the zinc initiator residue), the MALDI-TOF spectrum showed one set of signals corresponding to the polymer chains with hydroxyl and 2-amino-1-phenylethanolate end-groups (Figure S1). Thus, the data indicate that PCL was formed by the exclusive insertion of CL molecules into the Zn-O_alkoxide_ bond, and the Zn-C_alkyl_ bond remained intact. In the next step, following the developed polymerization procedure, the crude macromolecular organozinc EtZn(PCL-X) precursor was generated *in situ* in THF under an inert atmosphere and further used as a hybrid organozinc precursor of polymer-coated ZnO NCs (Figure 1). The stirred post-reaction mixture was exposed to air at ambient temperature, and the formation of poly(ϵ-caprolactone)-coated ZnO NCs (hereinafter denoted as ZnO-PCL) was monitored over a month using UV-Vis spectroscopy, photoluminescence (PL) spectroscopy, and high-resolution transmission electron microscopy (HRTEM; Figure 2). The kinetic analysis of reaction profiles obtained from *in situ* UV-Vis spectroscopy monitoring (Figures 2A and 2B) demonstrated that the macromolecular organometallic precursor reacts smoothly with moisture to initiate the nucleation process of zinc oxo species. The first detectable NCs appeared within 2–3 days, and the inorganic core diameter calculated from the Brus equation^50^ was 2.8 nm, 3.2 nm, and 3.3 nm after 3, 4, and 5 days of exposure to air, respectively (Figure 2C). Interestingly, over the following days, the absorption band and the maximum luminescence emission were only slightly shifted toward longer wavelengths, with the data revealing that the NCs reached a size of 4 nm after 30 days of exposure to air. Photoluminescence quantum yield measured in the solid state was found to be 21% and remained stable for weeks under air conditions. Time-resolved PL spectroscopy was applied to determine PL decay times for ZnO-PCL. The decay curve (Figure S2) was fitted with three exponential decays, namely fast components with a time constant of ca. 5 ns (48%), 52 ns (40%), and a slow component of ca. 1.4 μs (12%). The time-dependent HRTEM micrographs of ZnO-PCL are presented in Figures 2D and 2E. For example, after 5 days of exposure to air, the diameter of NCs was found to be 2.9 ± 0.4 nm (Figures 2D, S3, and S4). Further exposure to air resulted in a slight increase in the inorganic core diameter, and after 30 days the size of the NCs was 4.0 ± 0.4 nm (Figures 2E, S5, and S6). Thus, the HRTEM data corroborate well with the UV-Vis absorption data.

**Figure 3:**
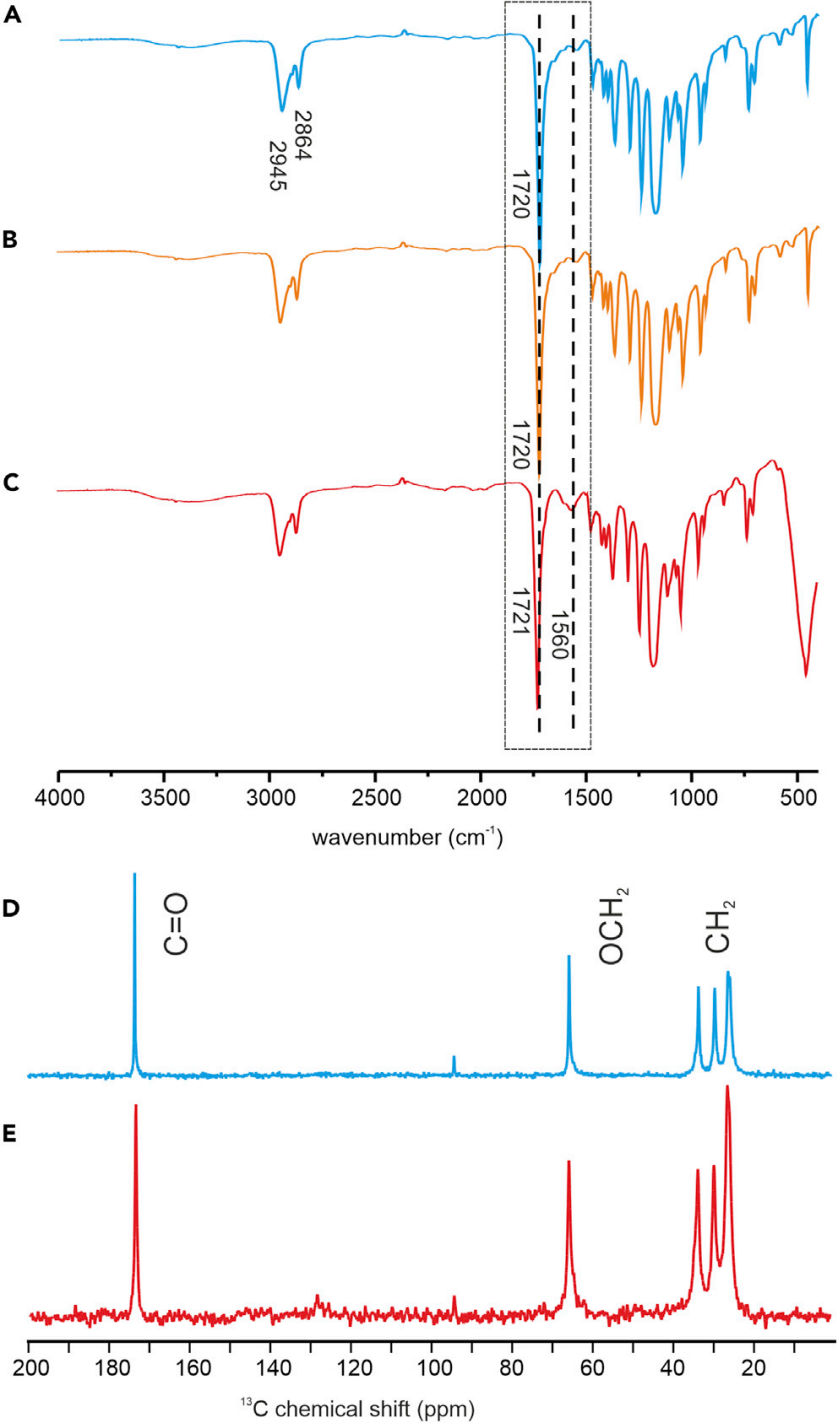
Spectral characterization of ZnO-PCL (A–C) The FTIR spectra of (A) PCL, (B) crude ZnO-PCL nanocomposite, (C) ZnO-PCL nanocomposite after purification. (D and E) ^13^C MAS NMR spectra of (D) PCL, (E) ZnO-PCL nanocomposite after purification.

The resulting THF colloidal mother solution of ZnO-PCL was stable, and no precipitation was noticed when allowed to stand up to one month at the ambient temperature. However, it should be stressed that the mother solution is comprised of both ZnO-PCL and free PCL (in the OSSOM procedure, the nucleation and growth process of X-type ligand-coated ZnO NCs is always accompanied by the liberation of an excessive amount of the proligand X-H^39^^,^^40^ and, accordingly, in the reported procedure, an excess amount of free PCL chains are liberated). Therefore, we subsequently attempted to isolate ZnO-PCL in a pure form from the mother solution using an anti-solvent precipitation method with hexane followed by centrifugation. The purification process was monitored with Fourier-transform infrared spectroscopy (FTIR). We note that the spectrum of neat PCL shows the characteristic bands of the C=O and C-O stretching vibrations of ester groups at 1720 cm^−1^ and 1293 cm^−1^, and the bands at 2945 and 2864 cm^−1^ corresponding to the CH_2_ group stretching vibration of the aliphatic backbone (Figure 3A). The positions of these bands remain unchanged in the case of as-synthesized ZnO-PCL nanocomposite (Figure 3B). Moreover, after multiple repetitions of the purification procedure, signals originating from PCL are still present in the FTIR spectrum (Figure S7). This feature highlights that the surface of the NCs is indeed functionalized by covalently bonded PCL chains. Remarkably, after washing away of excess polymer, the spectrum of solid ZnO-PCL material, apart from the characteristic bands of PCL, shows the fuzzy band in the range 3100–3600 cm^−1^ characteristic to the O-H stretch of water molecules and the band at 1560 cm^−1^ (Figure 3C). This signal might be attributed to carboxylate groups formed by the hydrolysis of polymer chains in the course of ZnO-PCL purification. The polymer-coated NCs were further investigated with ^13^C cross-polarization magic angle spinning (CP–MAS) NMR (Figures 3D and 3E). The spectrum of pure PCL shows a signal at 173 ppm that can be assigned to carbonyl groups from the polymer chain and signals at 65, 38, 29, and 25 ppm from the aliphatic chain. The same signals can be observed in the spectrum of ZnO-PCL. However, signals from ZnO-PCL are slightly broader than the respective signals of PCL, which supports the polymer bonding to NCs’ surface.

### Conclusions

In this work, we demonstrated a conceptually novel one-pot two-step method of the preparation of polymer-coated quantum-sized ZnO crystals. We showed that organozinc derivatives of aminoalcohols can be applied as initiators for the ring-opening polymerization of CL leading to the polyesters chains with a reactive Zn-R end functionality. This feature opened the unique possibility to combine two different processes: (i) the ring-opening polymerization mediated by an organozinc initiator and (ii) the self-supporting organometallic procedure involving the transformation of the resulting macromolecular RZn(polyester) precursor to ZnO NCs. This method allows obtaining ZnO NCs of uniform shape and small size distribution with polymer chains covalently anchored to NCs’ surface, which is resistant to being washed away even after multiple washing of the resulting nanocomposite. Moreover, the polymer shell firmly anchored to NCs’ surface allows for avoiding phase separation between ZnO and polymer in the solid state, which is crucial for NCs to be well-disperse in the polymer matrix. Research along these lines may pave the way for the design and nanoengineering of ZnO NCs coated by various polymer shells derived from the ring-opening polymerization of respective heterocyclic monomers.

### Limitations of study

In this work, we merged the initial ring-opening polymerization of ϵ-caprolactone mediated by an organozinc alkoxide initiator and an air-promoted transformation of the resulting macromolecular organozinc species, and H_2_O/O_2_ from the atmospheric air as the oxygen sources. Our experimental design was limited to both organozinc species as potential precursors of ZnO nanocrystals and the application of heterocyclic monomers that can undergo polymerization mediated by organozinc initiators.

## STAR★Methods

### Key resources table

**Table:** 

REAGENT or RESOURCE	SOURCE	IDENTIFIER
**Chemicals, peptides, and recombinant proteins**		

ε-Caprolactone	TCI	Cat# C0702
Diethylzinc	ABCR	Cat# AB355965
2-Amino-1-phenylethanol	Aldrich	Cat# A72405-10G

**Deposited data**		

Crystallographic data for EtZn-X structure	Cambridge Crystallographic Data Centre	CCDC 1907954

**Software and algorithms**		

InageJ	National Institutes of Health	https://imagej.nih.gov/ij/

### Resource availability

#### Lead contact

Further information and requests for resources and reagents should be directed to and will be fulfilled by the lead contact, Janusz Lewiński (janusz.lewinski@pw.edu.pl).

#### Materials availability

This study did not generate new unique reagents.

### Experimental model and subject details

This work did not need any unique experimental model.

### Method details

#### Materials

ε-Caprolactone was dried with CaH_2_ and distilled under reduced pressure. Diethylzinc was used as solution in dry hexane. All reactions involving air-sensitive reagents were conducted under an atmosphere of dry oxygen-free nitrogen gas using standard Schlenk technique.

#### Synthesis of ethylzinc 2-amino-1-phenylethanolate (EtZn-X)

To a THF solution (10 mL) of 2-amino-1-phenylethanol (1.0 mmol) diethylzinc in hexane (0.5 mL, 1.0 mmol) was added dropwise and stirred at −50°C (dry ice/isopropanol cooling bath) for several minutes. Then the reaction mixture was allowed to warm to room temperature and stirred for 2 h. Colorless crystals of ethylzinc 2-amino-1-phenylethanolate were obtained from THF/hexane mixture crystallized at 0°C. The isolated yield: 85%. ^1^H NMR measurement was performed on Varian Mercury 400 MHz spectrometer using C_6_D_6_ as a solvent. ^1^H NMR (C_6_D_6_, *δ*, ppm): 7-7.3 (m, 5H, C_6_**H**_5_); 5.27 (m, 1H, -C**H**(C_6_H_5_)OZn); 2.96 (m, 2H, C**H**_2_NH_2_); 2.31 (m, 2H, CH_2_N**H**_2_); 1.92 (m, 3H, -ZnCH_2_C**H**_3_); 0.38 (m, 2H, ZnC**H**_2_CH_3_).

##### Crystal structure determination of EtZn-X

The crystal was selected under Paratone-N oil, mounted on the nylon loops and positioned in the cold stream on the diffractometer. The X-ray data for EtZn-X were collected at 100(2) K on a Super-Nova Agilent diffractometer using Mo*Kα* radiation (λ = 0.71073 Å). The data were processed with *CrysAlisPro*, and solved by direct methods and refined using *SHELXL-2016/6*.^51^ All non-hydrogen atoms were refined with anisotropic displacement parameters. Hydrogen atoms were added to the structure model at geometrically idealized coordinates and refined as riding atoms. Crystal data for EtZn-X, C_30_H_45_N_3_O_3_Zn_3_: *M* = 691.08, crystal dimensions 0.18 × 0.12 × 0.06 mm^3^, monoclinic, space group *P* 21/c (no. 14), *a* = 12.0456(4) Å, *b* = 10.8978(4) Å, *c* = 23.8153(7) Å, *β* = 99.268(3) °, *U* = 3085.44(18) Å^3^, *Z* = 4, *F*(000) = 1440, *D*_c_ = 1.489 g cm^−3^, *T* = 100(2)K, *μ(*Mo-K*α*) = 2.348 mm^−1^, *θ*_max_ = 26.500°, 6143 unique reflections. Refinement converged at *R*1 = 0.0916, *wR*2 = 0.1318 for all data and 355 parameters (*R*1 = 0.0614, *wR*2 = 0.1131 for 4322 reflections with *I*_*o*_ > 2σ(*I*_*o*_)). The goodness-of-fit on F^2^ was equal 0.993. A weighting scheme *w* = [σ^2^(*F*_o_^2^ + (0.0418*P*)^2^ + 3.1964*P*]^−1^ where *P* = (*F*_o_^2^ + 2*F*_c_^2^)/3 was used in the final stage of refinement. The residual electron density = + 0.96/- 0.97 eÅ^−3^. CCDC 1907954. Figure S8 shows molecular structure of EtZn-X. Selected bond lengths [Å] and angles [°] are presented in Table S1.

#### Polymerization of ε-caprolactone

To a THF solution of EtZn-X (generated *in situ -* see “Synthesis of ethylzinc 2-amino-1-phenylethanolate (EtZn-X)”, 1.0 mmol) 1.06 mL (10.0 mmol) of CL was added. Polymerization was carried out for 5 h at ambient temperature. After 5 h essentially complete conversion of monomer was observed. Next THF was evaporated and methylene chloride was added in order to dissolve the product. The solution was shaken once with diluted hydrochloric acid to wash out the initiator residue. Then, the organic phase was washed with water three times and dropped into stirred hexane to precipitate the polymer. ^1^H NMR measurements were performed on Varian Mercury 400 MHz spectrometer using CDCl_3_ as a solvent. ^1^H NMR (CDCl_3_, *δ*, ppm), poly(ε-caprolactone): 4.06 (t, 2H, OC**H**_2_); 2.30 (t, 2H, OC(O)C**H**_2_); 1.64 (m, 4H, C(O)CH_2_C**H**_2_CH_2_C**H**_2_CH_2_O); 1.38 (m, 2H, C(O)CH_2_CH_2_C**H**_2_CH_2_CH_2_O).

##### Gel permeation chromatography (GPC)

Molar masses and molar mass distributions were determined with GPC instrument (GPC Max + TDA 305, Viscotek) equipped with Jordi DVB Mixed Bed columns (one guard and two analytical) at 30°C in dichloromethane (HPLC grade, Sigma-Aldrich) at flow rate of 1 mL/min with RI detection and calibration based on narrow PS standards (ReadyCal Set, Fluka). Results were processed with OmniSEC software (ver. 4.7). Number-average molecular weight (M_n_), weight-average molecular weight (M_w_) and molecular weight distributions M_w_/M_n_ (PDI) values determined by GPC were 2500 g/mol, 9300 g/mol and 3.9, respectively (Figure S9).

##### MALDI-ToF mass spectrometry

MALDI-ToF mass spectrometry was performed on Bruker UltrafleXtreme MALDI TOF Mass Spectrometer. *trans*-2-[3-(4-*tert*-Butylphenyl)-2-methyl-2-propenylidene]malononitrile (DCTB) or 2-(4-hydroxyphenylazo)benzoic acid (HABA) were used as MALDI matrix.

#### Preparation of poly(ϵ-caprolactone)-coated ZnO NCs (ZnO-PCL)

To a THF solution (5 mL) of 2-amino-1-phenylethanol (0.5 mmol) diethylzinc in hexane (0.25 mL, 0.5 mmol) was added dropwise and stirred at −50°C (dry ice/isopropanol cooling bath) for several minutes. Then the reaction mixture was allowed to warm to room temperature and stirred for 2 h. Next 0.53 mL (5.0 mmol) of CL was added. After 24 h, 30 mL of THF was added and reaction mixture was stirred while exposed to air at ambient temperature.

Purification procedure: To 20 mL of NCs in THF, 20 mL hexane was added to precipitate the NCs then the mixture was centrifuged at 13,000 rcf for 15 min. Separated NCs were dissolved in 20 mL THF and procedure was repeated three times.

#### Characterization of ZnO-PCL

##### Transmission electron microscopy

Size, shape and morphology of the nanocrystals were examined by High-Resolution Transmission Electron Microscopy (HRTEM). Nanocrystals samples were drop-cast (tetrahydrofuran solution) onto 300-mesh, holey carbon-coated copper grids (Quantifoil). Afterward, the excess solvent evaporated at room temperature. Nanocrystals samples were imaged using a C_s_ corrected scanning transmission electron microscope (STEM, HITACHI HD2700, 200 kV). The observations were carry on in three modes: SE (images used to study morphology), HAADF STEM (Z-contrast) and HR TEM (images showing the atomic structure). A wide variety of magnifications (from ×1500 up to ×8,000,000) were used to study the microstructure of ZnO samples. The size of nanocrystals was calculated by image analyses, using ImageJ software. For image analyses, a population of 100 crystals was used for each sample.

##### UV-vis absorption and photoluminescence

Absorption analysis was carried out by using a Hitachi U-2910 spectrophotometer, with solvents as reference. Photoluminescence spectra were recorded by using a Hitachi F-7000 fluorescence spectrometer. For PL measurement, an excitation wavelength λ_ex_ = 320 nm was used.

##### Photoluminescence quantum yield

Photoluminescence quantum yield (PLQY) values for solid state samples were measured with an absolute PLQY system (Quantaurus-QY Absolute PL quantum yield spectrometer C11347-11, Hamamatsu).

##### Photoluminescence decay

Photoluminescence decays were recorded using a Quantaurus-Tau fluorescence lifetime measurement system (C11367-11, Hamamatsu Photonics) equipped with the LED light source and photon counting measurement system. The samples were excited at 340 nm and PL was collected at 550 nm. The measurements were recorded using a pulse repetition rate at 20 kHz The data were collected with 1000 counts at the peak within 10 μs time range. The data were analyzed by a least-squares reconvolution procedure using the software package provided by Hamamatsu. The goodness of fit was judged in terms of χ2 value (lower than 1.3) and residuals distribution.

##### ^13^C cross-polarization magic angle spinning nuclear magnetic resonance

^13^C CP MAS NMR spectra were carried on an 11.7 T Bruker 500 Avance II spectrometer (Rheinstetten, Germany) equipped with a 4 mm broadband H/X probe head. Rototec-Spintec GmbH 4 mm Zirconia rotors with Kel-F caps were used. The frequency of the sample spinning was 10 kHz for all ^13^C CP MAS NMR experiments, which were performed at 298 K. All ^13^C CP MAS NMR spectra were referenced to glycine as an external standard.

##### Fourier transform infrared spectroscopy

FTIR spectra were recorded on a Bruker Tensor II spectrometer using the attenuated total reflection (ATR) mode. For each spectrum, 16 consecutive scans with a resolution of 4 cm^−1^ were averaged.

##### Dynamic light scattering

DLS measurements (Figures S10 and S11) were carried out using a Malvern Zetasizer Nano-ZS. All experiments were performed at 25°C. The samples were filtered through 0.45 mm membrane filters prior to analysis.

##### Powder X-Ray diffraction

Powder XRD data (Figure S12) were collected on a Empyrean diffractometer (PANalytical). Measurements employed Ni-filtered Cu Kα radiation of a copper sealed tube charged with 40 kV voltage and 40 mA current and Bragg-Brentano geometry with beam divergence of 1 deg. in the scattering plane. Diffraction patterns were measured in the range of 10–80 degrees of scattering angle by step scanning with step of 0.017°.

##### Thermogravimetric analysis

TGA measurements (Figure S13) were carried out by using a SDTQ600 thermal analyzer at a heating rate of 5°C/min under dynamic atmosphere of artificial air (80% nitrogen and 20% oxygen mixture) with gas flow at 100 mL/min.

### Quantification and statistical analysis

The size of NCs was calculated by image analyses, using ImageJ software. For image analyses, a population of 100 NCs was used for each sample.

### Additional resources

Our study has not generated or contributed to a new website/forum or not been part of a clinical trial.

## Data Availability

• Crystal structure of EtZn-X data have been deposited at Cambridge Crystallographic Data Centre (CCDC) and are publicly available as of the date of publication. Accession number is listed in the key resources table. • All data reported in this paper will be shared by the lead contact upon request. • This paper does not report original codes. • Any additional information required to reanalyze the data reported in this paper is available from the lead contact upon request.
