# Supplementary material for: A modular design approach to polymer-coated ZnO nanocrystals
Source: iScience. 2022 Dec 14;26(1):105759. doi: 10.1016/j.isci.2022.105759 (PMC9830196; doi:10.1016/j.isci.2022.105759)
Supplement: Document S1. Figures S1–S13 and Table S1 [file mmc1.pdf]

iScience, Volume *26*

Supplemental information

A modular design approach

to polymer-coated ZnO nanocrystals

Elżbieta Chwojnowska, Justyna Grzonka, Iwona Justyniak, Tomasz Ratajczyk, and Janusz Lewiński

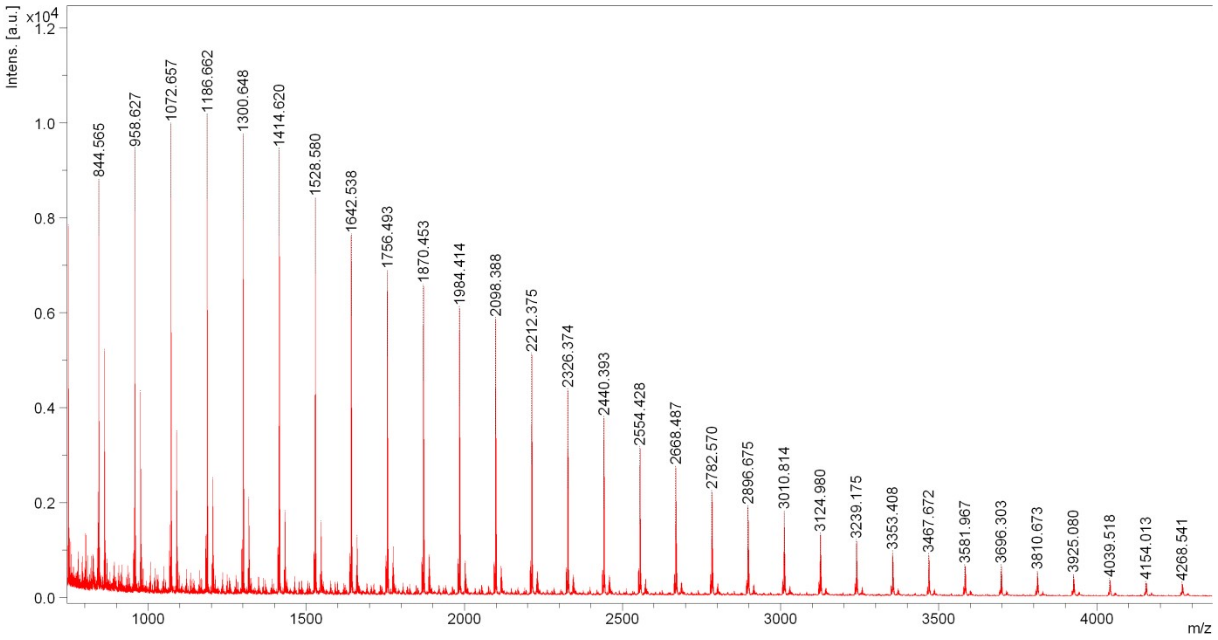

Figure S1. MALDI-ToF of poly(ε-caprolactone). Related to STAR Methods.

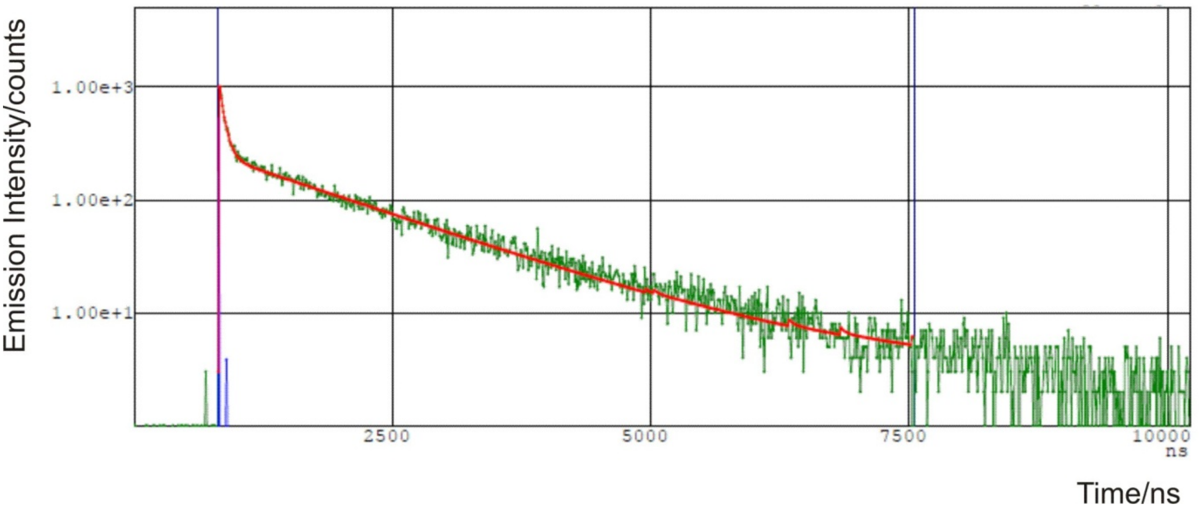

Figure S2. Decay curve of the emission intensity (green) and best fit using a multiexponential law conducted (red). The best fitting of decay curve (χ^2=1.041) enabled three different contributions to be distinguished, leading to lifetimes of τ_1= 4.70 ns; τ_2= 52.01 ns; τ_3= 1381.18 ns. Related to STAR Methods.

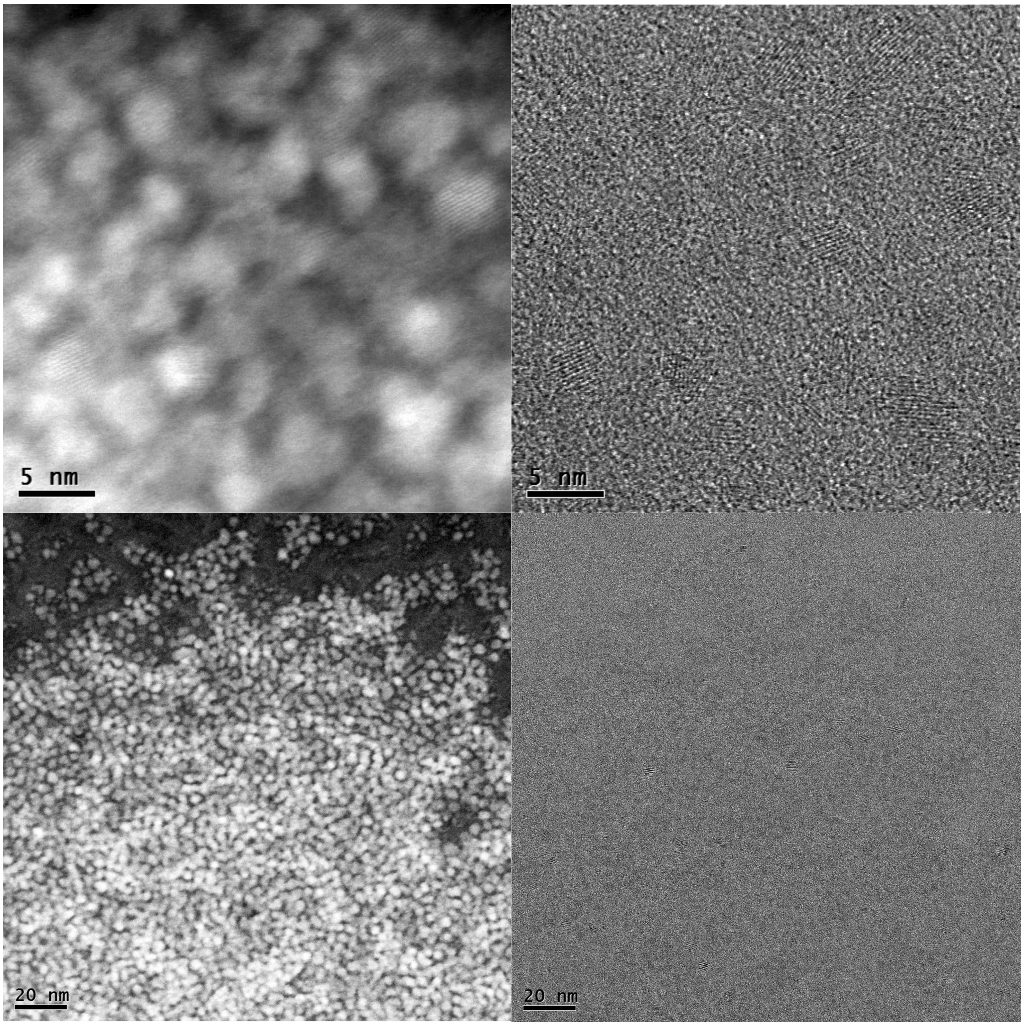

Figure S3. TEM images (modes: HAADF STEM left, HR TEM right) of **ZnO-PCL** after 5 days of exposure to air. Related to Figure 2.

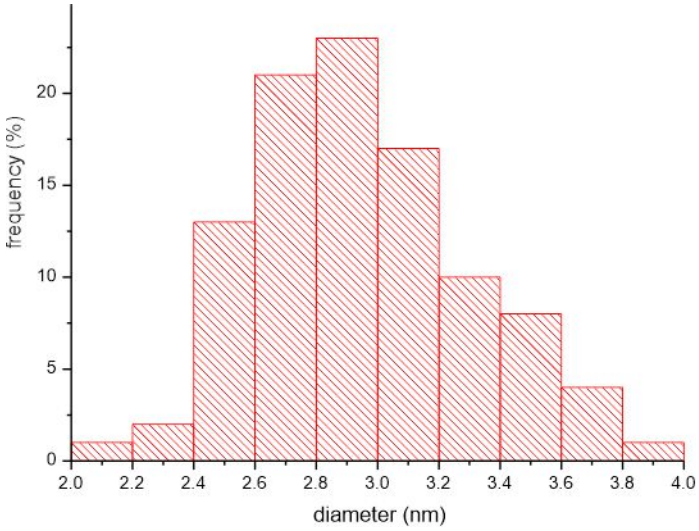

Figure S4. Size distribution of **ZnO-PCL** after 5 days of exposure to air, measured from TEM images. Related to Figure 2.

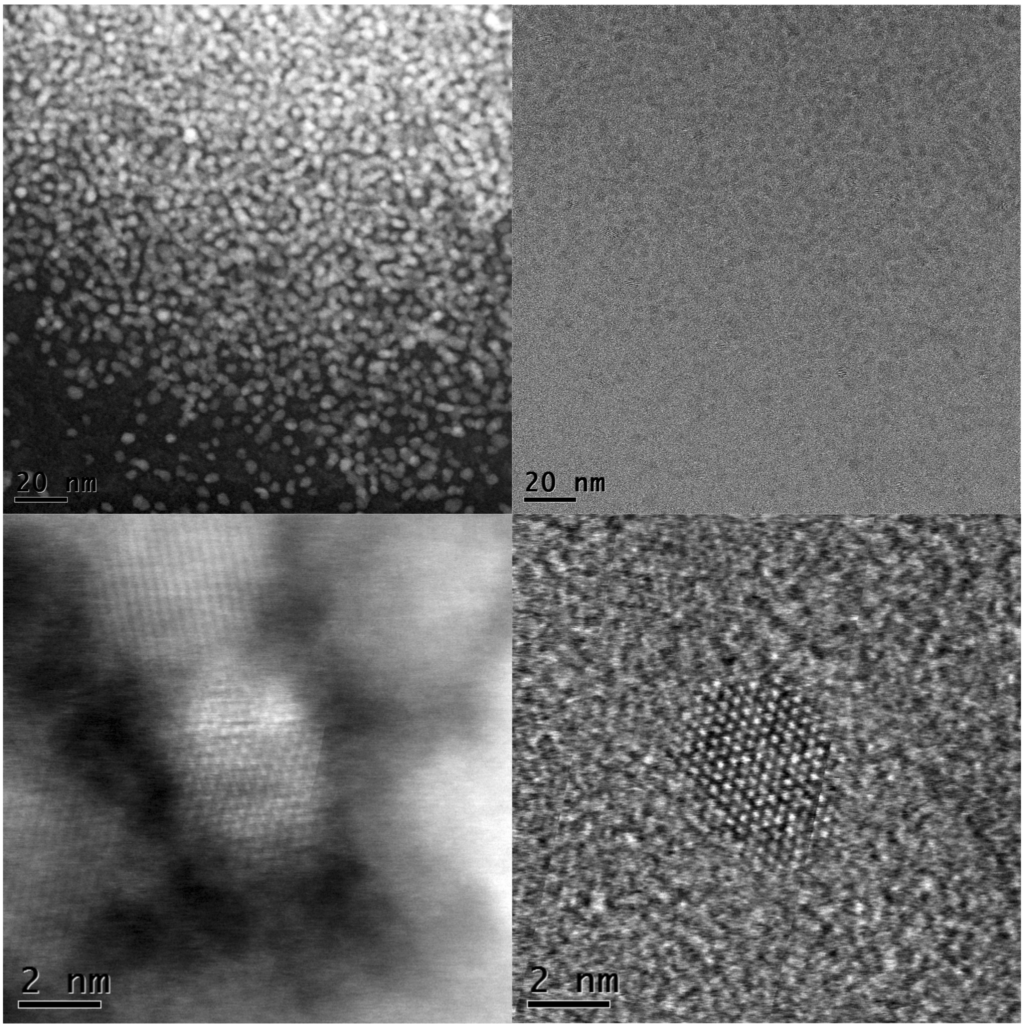

Figure S5. TEM images (modes: HAADF STEM left, HR TEM right) of **ZnO-PCL** after 30 days of exposure to air. Related to Figure 2.

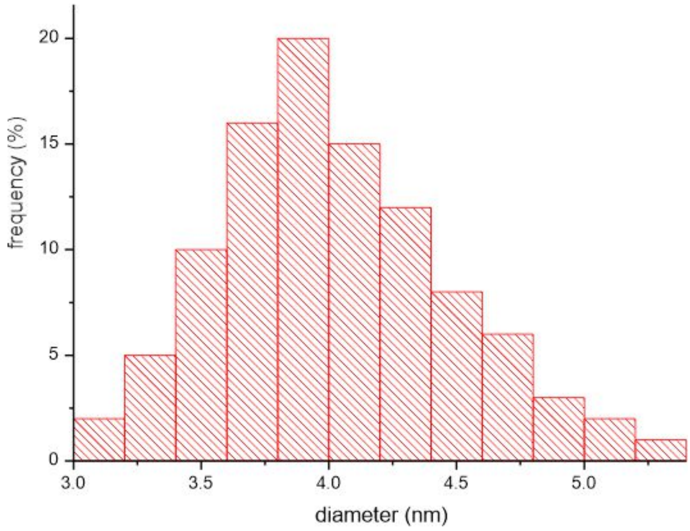

Figure S6. Size distribution of **ZnO-PCL** after 30 days of exposure to air, measured from TEM images. Related to Figure 2.

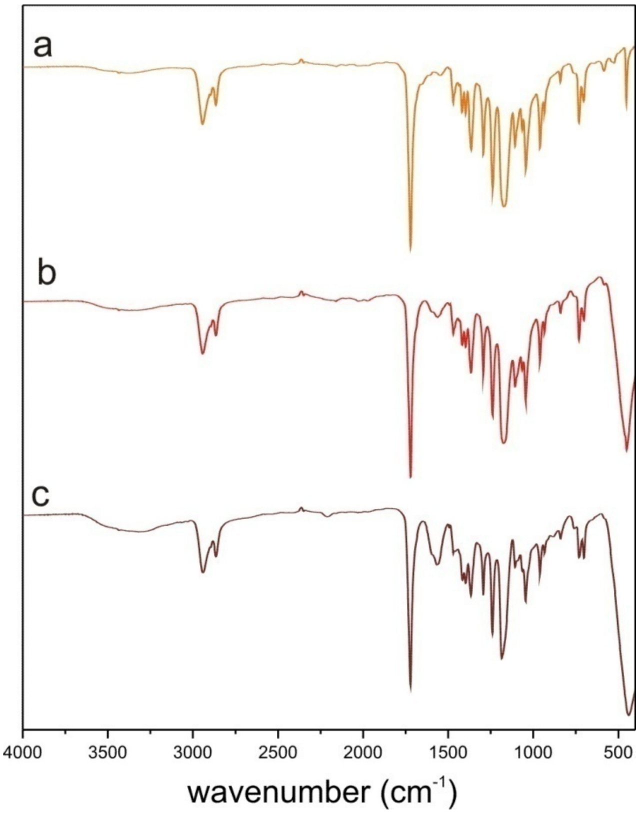

Figure S7. FTIR of crude **ZnO-PCL** (a), **ZnO-PCL** nanocomposite after purification (b); **ZnO-PCL** nanocomposite after multiple repetitions of the purification procedure (c). Related to Figure 3.

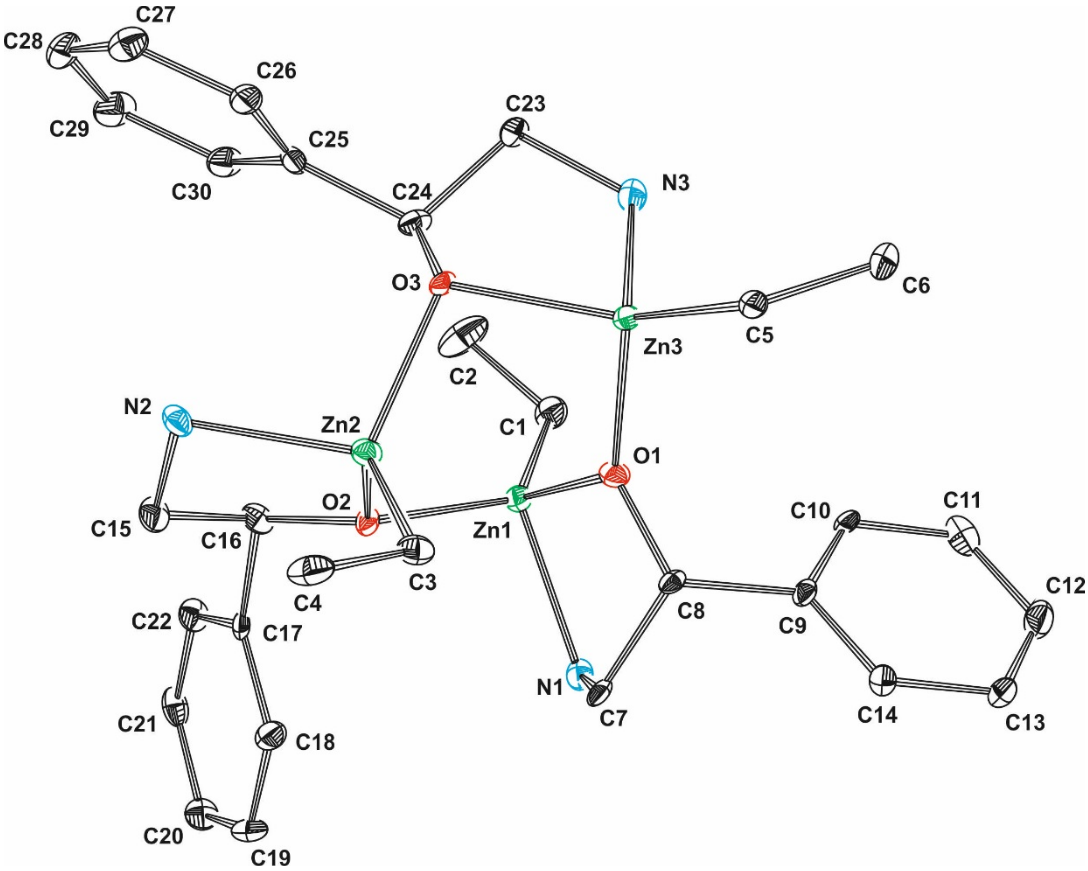

Figure S8. Molecular structure of ethylzinc 2-amino-1-phenylethanolate (**EtZn-X**). All hydrogen atoms have been omitted for clarity. Related to STAR Methods.

Table S1. Selected bond lengths [Å] and angles [°] for **EtZn-X**. Related to STAR Methods.

Bond lengths		Angles	
Zn1 – O1	2.021(3)	O1 – Zn1 – O2	91.91(13)
Zn1 – O2	1.987(3)	O1 – Zn1 – N1	82.62(14)
Zn1 – N1	2.134(4)	O1 – Zn1 – C1	122.15(17)
Zn1 – C1	1.983(5)	N1 – Zn1 – C1	120.09(19)
Zn2 – O2	2.036(3)	O2 – Zn1 – C1	131.10(19)
Zn2 – O3	2.002(3)	O2 – Zn1 – N1	96.23(15)
Zn2 – N2	2.127(4)	O2 – Zn2 – O3	104.60(13)
Zn2 – C3	1.981(5)	O2 – Zn2 – N2	81.68(14)
Zn3 – O1	2.003(3)	O2 – Zn2 – C3	124.78(17)
Zn3 – O3	2.012(3)	N2 – Zn2 – C3	120.9(2)
Zn3 – N3	2.135(4)	O3 – Zn2 – C3	115.72(16)
Zn3 – C5	1.972(5)	O3 – Zn2 – N2	102.98(15)
		O1 – Zn3 – O3	94.44(13)
		O1 – Zn3 – N3	99.18(15)
		O1 – Zn3 – C5	118.77(17)
		N3 – Zn3 – C5	120.81(17)
		O3 – Zn3 – C5	131.32(17)
		O3 – Zn3 – N3	83.53(15)

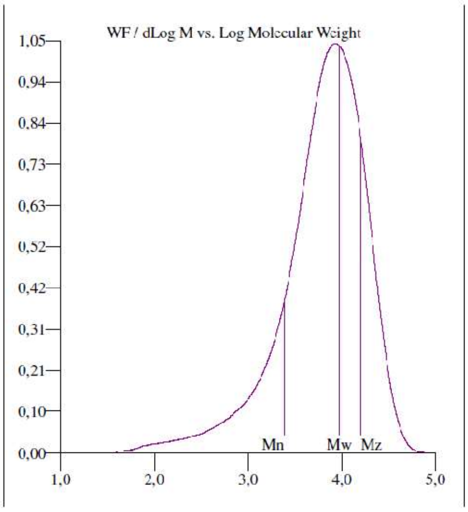

Figure S9. GPC of poly(ε-caprolactone). Related to STAR Methods.

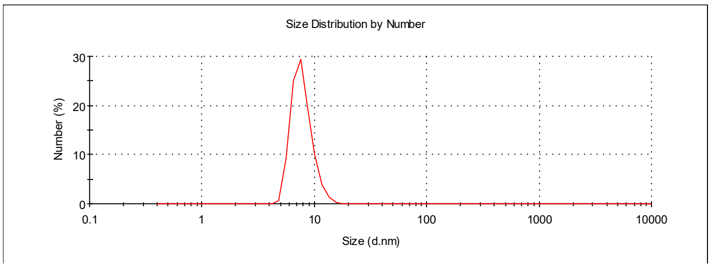

Figure S10. DLS in THF of **ZnO-PCL** after 5 days of exposure to air. Related to STAR Methods.

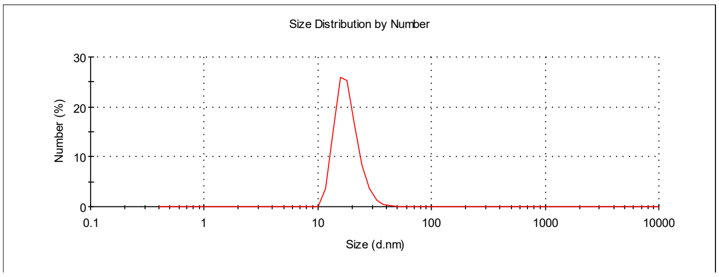

Figure S11. DLS in THF of **ZnO-PCL** after 30 days of exposure to air. Related to STAR Methods.

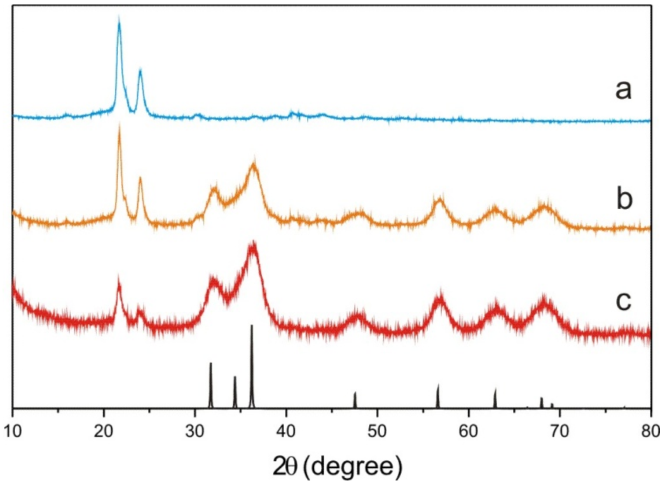

Figure S12. PXRD pattern of PCL (a), crude **ZnO-PCL** nanocomposite (b), **ZnO-PCL** nanocomposite after purification(c). Related to STAR Methods.

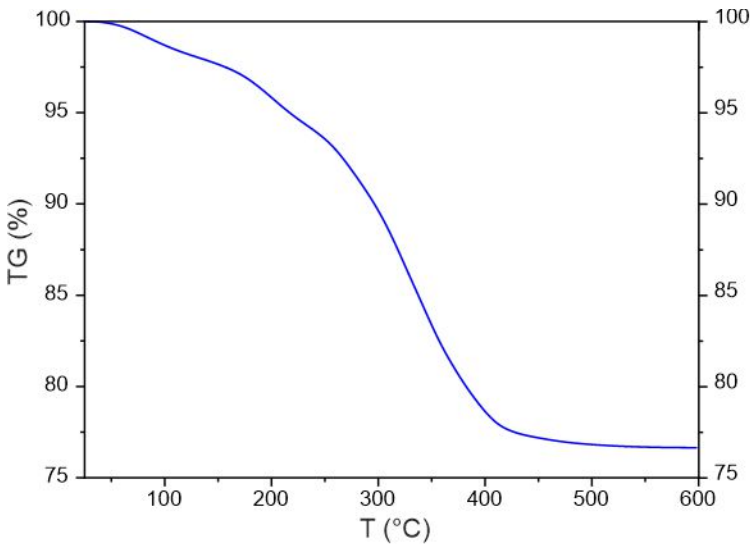

Figure S13. Thermogravimetric analysis showing the decomposition of **ZnO-PCL** in an air atmosphere. Related to STAR Methods.
